# Genome-Wide Mapping of Furfural Tolerance Genes in *Escherichia coli*

**DOI:** 10.1371/journal.pone.0087540

**Published:** 2014-01-28

**Authors:** Tirzah Y. Glebes, Nicholas R. Sandoval, Philippa J. Reeder, Katherine D. Schilling, Min Zhang, Ryan T. Gill

**Affiliations:** 1 Chemical and Biological Engineering, University of Colorado Boulder, Boulder, Colorado, United States of America; 2 National Bioenergy Center, National Renewable Energy Laboratory, Golden, Colorado, United States of America; University of Massachusetts, United States of America

## Abstract

Advances in genomics have improved the ability to map complex genotype-to-phenotype relationships, like those required for engineering chemical tolerance. Here, we have applied the multiSCale Analysis of Library Enrichments (SCALEs; Lynch et al. (2007) *Nat. Method*.) approach to map, in parallel, the effect of increased dosage for >10^5^ different fragments of the *Escherichia coli* genome onto furfural tolerance (furfural is a key toxin of lignocellulosic hydrolysate). Only 268 of >4,000 *E. coli* genes (∼6%) were enriched after growth selections in the presence of furfural. Several of the enriched genes were cloned and tested individually for their effect on furfural tolerance. Overexpression of *thyA*, *lpcA*, or *groESL* individually increased growth in the presence of furfural. Overexpression of *lpcA*, but not *groESL* or *thyA*, resulted in increased furfural reduction rate, a previously identified mechanism underlying furfural tolerance. We additionally show that plasmid-based expression of functional LpcA or GroESL is required to confer furfural tolerance. This study identifies new furfural tolerant genes, which can be applied in future strain design efforts focused on the production of fuels and chemicals from lignocellulosic hydrolysate.

## Introduction

Genome engineering strategies are limited by the massive combinatorial search space created when multiple genetic units must be optimized in tandem [1], [2]. While early efforts focusing on engineering a small number of genetic parts have resulted in several impressive results [3]–[5], efforts focused on the engineering of complex phenotypes have remained a key challenge for the field. This challenge is especially true when the genetic bases of the targeted phenotypes are poorly understood, as is the case for many tolerance phenotypes [1], [6]–[10].

Advances in methods for mapping genotype-to-phenotype relationships have helped address this issue ([11]–[17] for a detailed review see [18]). Mapping approaches enable rapid identification of novel gene targets for strain design. These strategies generally employ well-defined libraries that allow for tracking of all members in parallel during a high-throughput screen or selection. Importantly, multiplex genome-modification strategies can be used to then develop combinatorial mutants of multiple alleles identified during genome mapping [19]–[22]. Together, these strategies represent an approach for rationally searching genetic space during genome engineering efforts [1].

Here, we have applied one of these new methods for genome mapping, multiSCale Analysis of Library Enrichments (SCALEs) [13], to engineer furfural tolerance, an important phenotype for improving microbial biofuel production from lignocellulosic hydrolysate. Lignocellulosic biomass (e.g., switchgrass and corn stover) is a proposed feedstock for next-generation biofuel production [23], since it is a renewable and sustainable source of sugars (from hemicellulose and cellulose). Biomass pretreatment and saccharification release sugars into the liquid hydrolysate, which can be fermented into biofuels, but also release a variety of inhibitory compounds. Furfural, is a heterocyclic aldehyde formed from pentose degradation during pretreatment, and is one of the key inhibitory compounds in hydrolysate ([24] for a review on hydrolysate toxicity see [25]).

Furfural is a known DNA mutagen in *Escherichia coli* [26]–[28]. In addition, growth inhibition induced by furfural has been linked to the reduction of furfural to furfuryl alcohol by NADPH-dependent oxidoreducatses [29]. This reduction elicits a variety of negative responses in the cell, causing starvation of available NADPH necessary for biosynthetic processes such as sulfur assimilation [30] and pyrimidine synthesis necessary for DNA repair [31]. Alleviation of NADPH-starvation can be obtained by silencing NADPH-dependent oxidoreductases [32], increasing NADH-dependent reductase expression [33] and activity [34], increasing expression of a predicted oxidoreductase [35], and overexpressing the NADPH-restoring transhydrogenase PntAB [30]. A recent study combined many of these mutations together to improve production of ethanol and succinate from hydrolysate [36]. Similar toxicity mechanisms and genetic manipulations have been beneficial for engineering *E. coli* for tolerance to 5-hydroxymethylfurfural [37], a hexose degradation product in hydrolysate. In addition to directly redox related mechanisms, reactive oxygen species (ROS) accumulation has been observed in yeast cells [38] and *E. coli* [39] when treated with furfural, which is a common phenotype associated with DNA damage [40], as well as more generally with chemotoxicity [41].

We hypothesized that use of the SCALEs method would identify additional novel targets for engineering furfural tolerance. SCALEs employs four genomic libraries, each with distinct insert sizes (1, 2, 4 or 8 kb) to test, in parallel, the effect increased dosage of insert sequence (containing gene(s) and/or operon(s)) has under a selective pressure. Individual clone frequencies are calculated using microarray technology and the SCALEs signal processing algorithms, as described by Lynch et al. [13]. The multiscale analysis algorithm assigns the microarray signals according to the contribution from each library size. This method produces genome-wide fitness data at approximately 125 nucleotide resolution, thus allowing for precise mapping of the genetic basis of high fitness clones. The SCALEs method has previously been used to map genotype-to-phenotype relationships in a variety of applications, including: engineering tolerance to anti-metabolites [42], solvents [8], [9], [43], organic acids [7], [44], [45], antibiotics [46], [47], as well as identifying genes restoring redox balance [48]. Here, we applied the SCALEs method to simultaneously map furfural related fitness effects resulting from overexpression of all *E. coli* genes (a total of >10^5^ individual clones were evaluated). Follow-up studies confirmed novel furfural tolerance genes.

## Materials and Methods

### Bacteria, plasmids, and media

*E. coli* BW25113 Δ*recA*::Kan was obtained from the Keio Collection [49], and the kanamycin resistance cassette was removed according to the previously designed protocol [50] to yield BW25113 Δ*recA*::FRT, which was used as the host for all studies here, as similarly reported [7], [9]. The pSMART-LCK (Lucigen) vector was used for library and clone construction. Ligated vector with no insert was used as the control. All cultures were grown at 37°C. Kanamycin was used where appropriate (30 µg ml^−1^). Selections and growth tests were performed in MOPS minimal medium [51] with 0.2 w v^−1^% glucose. Luria-Bertani (LB) medium was used for routine applications.

### Genomic libraries, selection, and microarray analysis

Genomic libraries were prepared previously by Warnecke et al. [45], by extracting genomic DNA from *E. coli* K-12 (ATCC #29425) to construct 1, 2, 4, and 8 kb SCALEs libraries in pSMART-LCK. Plasmid libraries were extracted from originally prepared cells with a Plasmid Midi Kit (Qiagen) and freshly transformed into the BW25113 Δ*recA*::FRT host. Samples of the transformants were diluted to confirm a minimum of 10× 99% library coverage (>10^5^ cells) [13]. After a one hour recovery following transformation, the libraries were diluted into a single MOPS minimal medium culture and grown to early exponential phase. Aliquots of 50 µl were spread onto 20 MOPS minimal medium plates (control) or MOPS minimal medium plates with 0.75 g l^−1^ furfural (>10^5^ cells total plated for each condition). Plates were incubated until growth appeared (one day for control plates and three days for furfural plates). Cells were harvested from the plates and plasmids were extracted with a Plasmid Midi Kit (Qiagen). Samples were digested and prepared for microarray analysis according to the method of Lynch et al. [13]. Analysis of the resulting data file was performed with the SCALEs software [13] as previously described [7], with plasmids from minimal medium plates without furfural serving as the control sample. Fitness, *W*, is calculated for an individual clone, *i*, by *W  =  frequency_i,furfural_/frequency_i,control_*. Because overlapping clones may contain part of all of a particular gene, individual gene fitness scores were calculated as a summation of clones containing a given gene, weighted by the fraction of the gene contained in the clone. Analysis of Gene Ontology term enrichment was performed with the Batch Genes tool available on the GOEAST website [52] using default settings.

### Clone construction

Primers for gene amplification were designed to amplify the native promoter and open reading frame for each target and are listed in Table S1. Phosphorylated cassettes were ligated into pSMART-LCK according to manufacturer directions and then transformed into electrocompetent cells. Plasmid constructs were confirmed by gel electrophoresis and sequencing.

### Growth curves and plating assays

Cultures inoculated from freezer stocks were grown overnight in LB medium. Seed cultures were inoculated with 2 v v^−1^% overnight cultures into MOPS minimal medium, grown into exponential phase, and diluted to OD_600_ 0.195–0.200 to be used as innocula for test cultures at 10 v v^−1^%. Growth curve studies were performed in 15 ml conical tubes with 5 ml liquid volume. Furfural was added to a concentration of 0.75 g l^−1^. Growth was monitored at 600 nm for 24 hours (n = 3).

For plating assays, normalized seed cultures were diluted by half, from which 1 µl (∼10^4^ cells) was streaked onto MOPS minimal medium plates with furfural (0–1.5 g l^−1^). Plates were incubated at 37°C for up 72 hours.

### Furfural reduction measurements

Furfural was measured with a spectrophotometer at 284 nm [53]. A standard curve was prepared in MOPS minimal medium and fit by linear regression. Standards and samples were diluted 1∶1000 in water. Samples were collected from growth curve cultures during cell density measurements and stored at 4°C for a maximum of 12 hours prior to analysis. Furfural measurements were normalized to cell density, and reduction rate was calculated from the regression line during the transition from lag phase to exponential phase, where reduction trends were linear. Samples were collected over 24 hours, at which point furfural was no longer observed in the cultures.

### Mutation frequency analysis

Mutation frequency was measured by proxy with frequency of rifampin resistance [54], [55]. Cell cultures were grown overnight, harvested by centrifugation, diluted 10-fold into 25 ml of MOPS minimal medium, and incubated for 30 minutes to allow for growth to begin. Furfural was added to 0.75 g l^−1^ and cultures were incubated for 3 hours. Cells were then harvested and diluted accordingly for measuring total CFU count (LB agar) and spontaneous mutants (LB agar with 100 µg ml^−1^ rifampin). Mutation frequency was calculated by dividing the number of rifampin resistant mutants by total CFU (n = 4).

### qPCR expression analysis

Strains were prepared and grown according to the same procedure used for growth curve analysis with the following exception: strains were inoculated into MOPS minimal medium without furfural and grown for 6.5 hours (into exponential phase). Aliquots of 1 ml were harvested by centrifugation, decanted, and immediately frozen in a dry ice-ethanol bath, and stored at −80°C until further use. For RNA extraction, 400 µl of RNAProtect Cell Reagent (Qiagen) was added to pellets, mixed by pipetting, and then processed with an RNAEasy Mini Kit (Qiagen). RNA samples were analyzed with an iTaq Universal SYBR Green One-Step Kit (Bio-Rad). Expression of *cysG* was used as a housekeeping reference gene [56] for calculating relative fold-change (n = 2–3).

### Site-directed mutagenesis clone construction and testing

Mutants were constructed using a QuikChange Lightning Kit (Agilent Technologies) according to manufacturer's instructions with either the *lpcA* or *groESL* pSMART-LCK construct (Lucigen) as the template. Primers were designed to introduce point mutations as follows: *lpcA*(E65Q) using TGCACTTTGCCGAACAGTTGACCGGTCGCTACCG and its complement sequence; *groES*(M1R) using CTCAAAGGAGAGTTATCACGGAATATTCGTCCATTGCATGATCG and its complement sequence; and *groEL*(M1R) with AAGGAATAAAGATACGGGCAGCTAAAGACG and its complement sequence. Growth studies were prepared as done for growth curve analysis, with the OD_600_ readings measured at 20 hours. Percentage improvement, compared to blank vector control, was used for comparison of the clones (n = 3).

### Statistical analyses

Sample averages were calculated for all phenotypic analyses and are plotted and reported with ± one standard error. Student's t-test was used to calculate one-tailed *p*-values. Values are reported within the text with ± one standard error.

## Results and Discussion

### Application of SCALEs method to identify furfural tolerance genes

**Figure 1:**
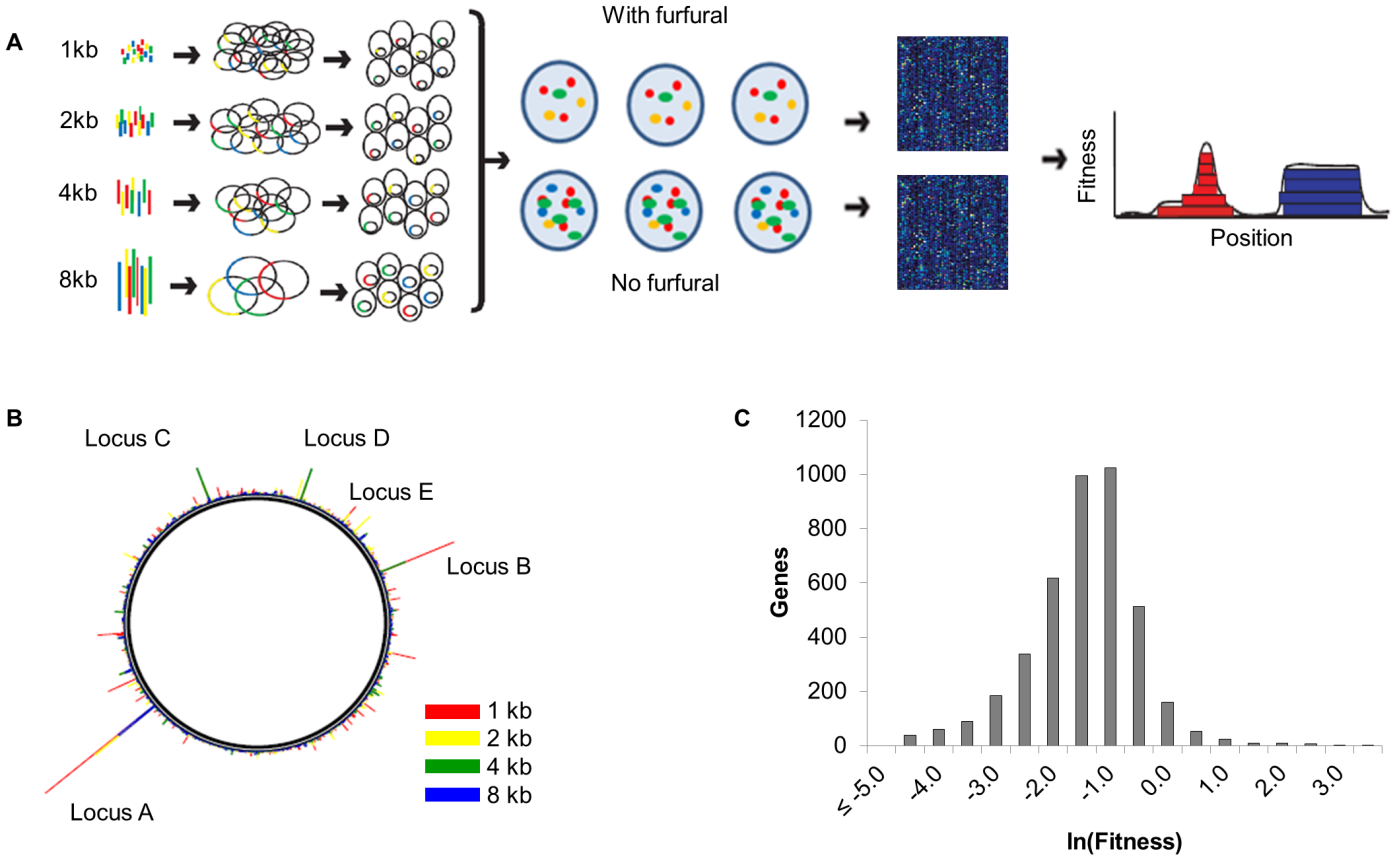
Overview of furfural selection and SCALEs analysis. A) 1, 2, 4, and 8 kb fragments were prepared from *E. coli* genomic DNA and ligated into pSMART-LCK vector. Each sized genomic library was transformed into BW25113 Δ*recA*::FRT host cells, recovered, mixed together, and then grown on minimal medium plates (control) or solid minimal medium with 0.75 g l^−1^ furfural. Cells were harvested from the plates and microarrays (square boxes) were run with plasmid extracts from both the furfural and control plates in order to determine individual gene fitness scores (*W*). The fitness vs. position plot illustrates how different clones (stacked rectangles) can contribute to an individual gene's fitness. The red “triangle” has contribution from various sized clones, but is centered around a specific locus, whereas the blue “rectangle” represents a high fitness score from the presence of one single sized clone (e.g., requiring a large operon where smaller library sizes would not be found). B) Genome plot depicting clone fitnesses for the different library sizes. Loci corresponding to the top gene fitness scores are labeled. C) Histogram of log-transformed gene fitness scores, where increased fitness corresponds to ln(*W*)>0.

SCALEs libraries containing >10^5^ clones were selected on solid minimal medium with 0.75 g l^−1^ furfural (Fig. 1A). Libraries cultured on minimal medium plates with no furfural served as the control in order to account for growth on minimal medium alone. The selection was performed on plates to provide a microenvironment where clones were spatially isolated, in an effort to remove population effects (e.g., decreased local furfural concentration due to increased reduction by certain clones) that might interfere with assessing individual clone fitness [31]. Colonies were harvested from plates after growth appeared (one day for control and three days for furfural treatment) and plasmids were extracted and analyzed with microarrays to determine clone concentration at approximately 125 bp resolution (Fig. 1B). A fitness score was calculated for each gene to determine those that were differentially enriched with furfural selection. High-fitness genes were identified across the entire genome and more than one size of library insert contributed to loci with the highest fitness scores (see Fig. S1 for details).

A total of 268 genes, or ∼6% of all *E. coli* genes, were enriched through selection (Fig. 1C), indicating that a strong selective pressure was applied (all genes with increased fitness during furfural selection are provided in Table S2). Using the Batch Genes program [52], we analyzed the increased fitness genes by Gene Ontology (GO) terms and found that significantly enriched terms were primarily associated with cell membrane (e.g., enterobacterial common antigen) and wall (e.g., peptidoglycan) biosynthetic processes, suggesting that membrane and wall formation are important for furfural tolerance (Fig. S2). No cellular component or molecular function GO terms were significantly enriched.

### Confirmation of furfural tolerance

Based on the gene-specific fitness scores (Table S2), we determined that the top 19 genes mapped to only five distinct loci (labeled A–E, Fig. 1B). Visual inspection of the clone fitness patterns associated with each loci suggested specific genes that were the primary (or sole) contributor towards fitness (as shown in Fig. S1). We then constructed individual clones for each of the hypothesized fitness-contributing gene(s) from the top five loci (Table 1): locus A (*thyA*), locus B (*ybiY*), locus C (*groESL*), locus D (*lpcA*), and locus E (*ybaK*).

**Table 1:** Gene(s) cloned for confirmation studies.

Rank	Gene	Fitness	Locus	Function
2	*ybiY*	20.2	B	Predicted pyruvate formate lyase activating enzyme
6	*thyA*	14.8	A	Thymidylate synthase
8	*groEL*	13.5	C*	GroEL chaperone
12	*lpcA*	10.5	D	D-sedoheptulose 7-phosphate isomerase
17	*groES*	7.5	C*	GroES chaperone
19	*ybaK*	7.1	E	Cyc-tRNA^Pro^ and Cyc-tRNA^Cys^ deacylase

*groESL operon was cloned into a single plasmid.

**Figure 2:**
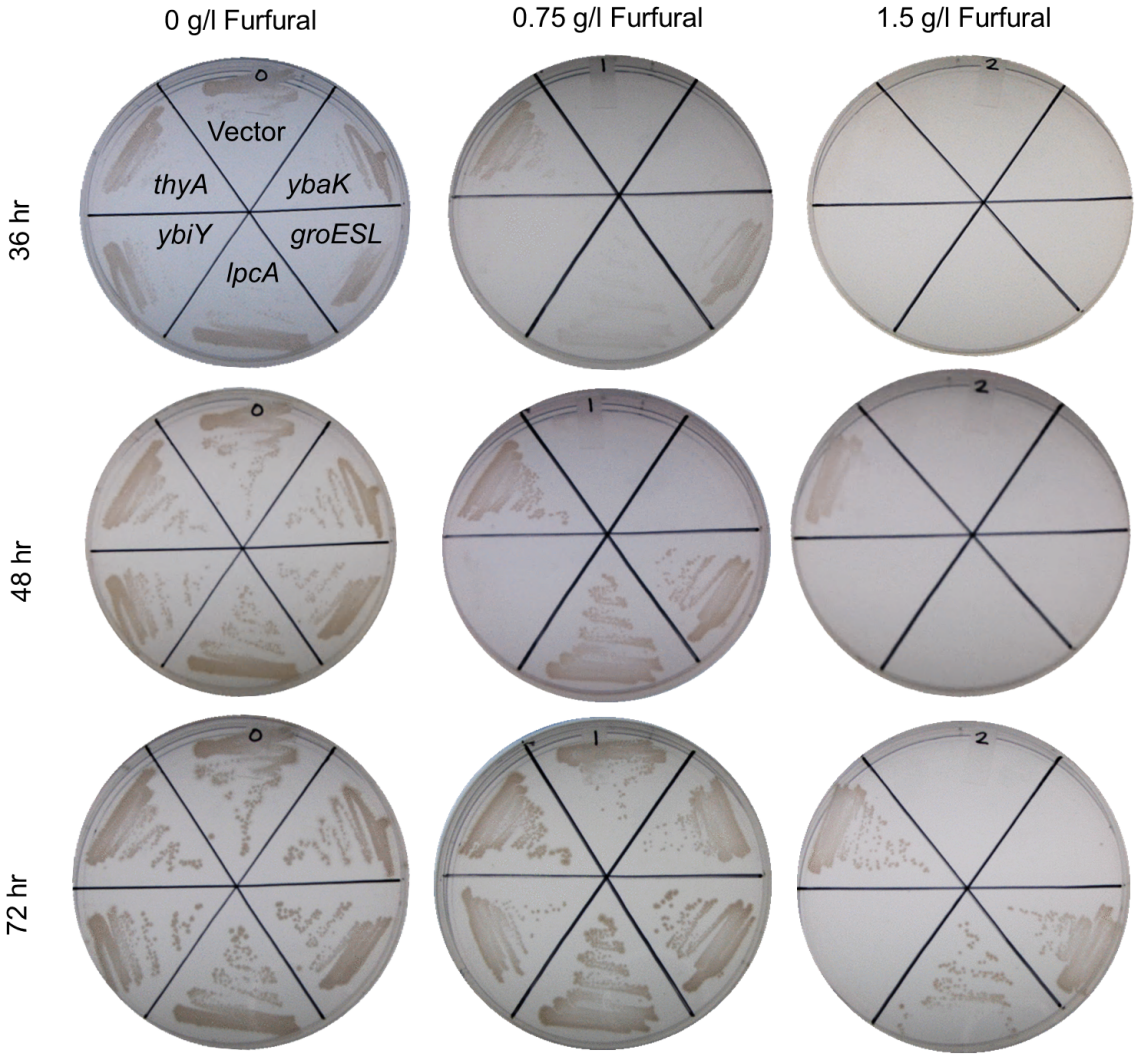
Plating assay of hypothesized tolerance-conferring clones identified in SCALEs selection. Cells (10^4^) were streaked onto solid minimal medium with 0, 0.75, or 1.5 g l^−1^ furfural (0, 1, or 2× selection pressure) and growth was observed for 72 hours.

We first attempted to confirm tolerance of the hypothesized fitness-contributing gene(s) under the same conditions used in our growth selections (i.e., improved growth on solid minimal medium with furfural). Cultures of each of the five clones were streaked onto solid medium supplemented with furfural at 0, 0.75 g l^−1^, or 1.5 g l^−1^ (corresponding to 0, 1 and 2× selection concentrations). Growth was monitored for three days, consistent with the time of furfural selection. At both furfural treatment levels, growth appeared first from *thyA*, followed by *lpcA* and *groESL* clones (Fig. 2). Clones overexpressing *ybiY* or *ybaK* were not observed to confer improved tolerance compared to vector control and were thus removed from further study. Based on our previous experience with SCALEs [7]–[9], [13], [42]–[48], we expect that the lack of observed tolerance phenotypes from *ybiY* and *ybaK* is likely due to these genes requiring other genes in the enriched loci, although we cannot eliminate the possibility that they were false positives [45].

**Figure 3:**
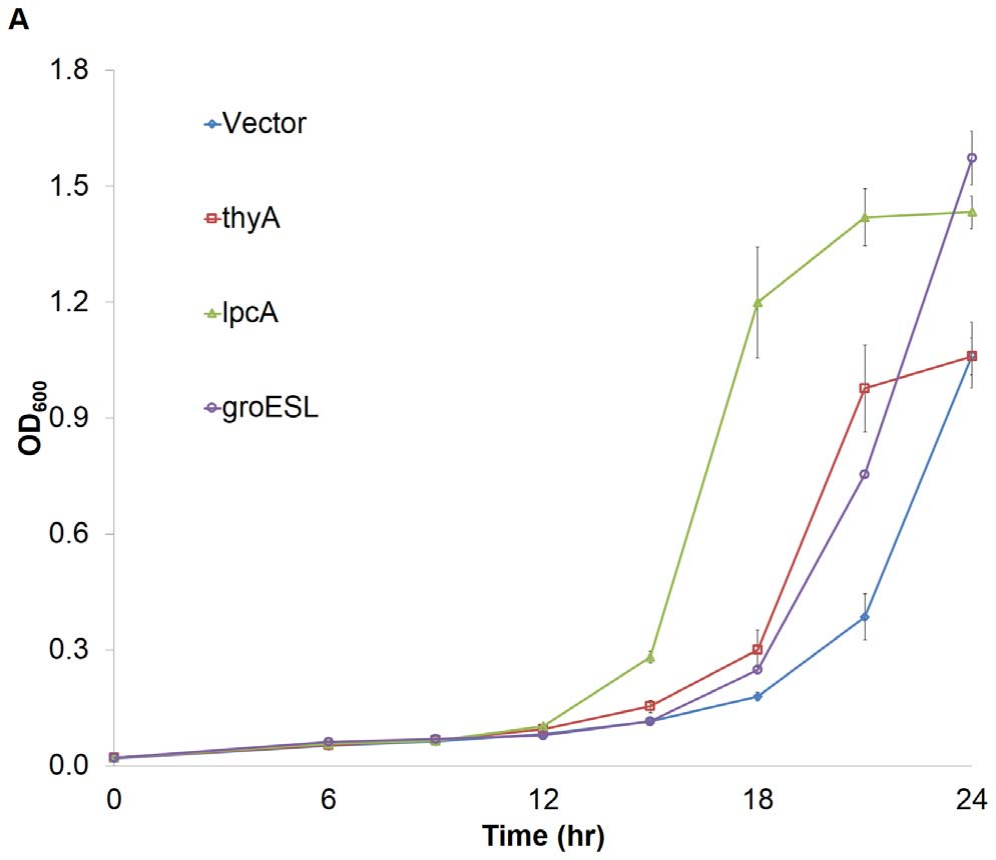
Growth curve analysis of tolerant clones grown in minimal medium with 0.75^−1^ furfural. A) Seed cultures were inoculated into furfural at the same initial density and grown for 24 hours. Optical density was recorded every 3–6 hours. Error bars represent one standard error (n = 3). Double asterisks denote *p*<0.05.

We next tested each confirmed tolerance clone for improved growth in planktonic cultures. Growth curves of *thyA*, *lpcA*, and *groESL* overexpression clones were performed and we observed improved growth from all three strains tested (Fig. 3). Interestingly though, *thyA*, which was the first strain with visible growth on the solid medium with furfural (Fig. 2), had a longer lag phase than the *lpcA* clone, which was the first clone to leave lag phase in planktonic cultures. Additionally, both the *groESL* and *lpcA* clones had higher density at 24 hours than the *thyA* clone or the empty vector control, at which point we stopped sampling due to the complete disappearance of furfural.

ThyA, LpcA, and GroEL-ES are involved in relatively distinct cellular processes. Thymidylate synthase, encoded by *thyA*, catalyzes the conversion of dUMP to dTMP during *de novo* pyrimidine biosynthesis. ThyA overexpression has previously been observed to confer furfural tolerance [31], presumably by increasing dTMP availability for increased DNA repair suspected to occur during furfural treatment.

The isomerase encoded by *lpcA* catalyzes the first committed step in lipopolysaccharide (LPS) core biosynthesis by routing a pentose phosphate pathway (PPP) metabolite, D-sedoheptulose 7-phosphate, towards heptose formation and subsequent incorporation into the inner core region of LPS. Functional LPS formation is widely documented as important for tolerance to hydrophobic compounds [57]–[59]. Also, the PPP is a major source of NADPH in *E. coli*, and increased upper pathway flux through this pathway (to make up for losses due to increased LPS synthesis) could lead to increased NADPH formation, limitations of which are thought to play an important role in furfural toxicity [29], [30], [32]–[36]. Previous studies for furfural tolerance targets have not previously identified *lpcA* or LPS formation, but previous SCALEs studies from our laboratory have identified *lpcA* as a highly enriched locus in acetate and ethanol selections, where *lpcA* overexpression was confirmed to improve ethanol tolerance several fold. [7], [9].

The GroEL-ES chaperonin complex, encoded by *groESL*, is essential for cell growth under a range of temperatures [60], is required for proper folding of some essential proteins [61], and is a well-known stress associated protein [62]–[64]. Moreover, overexpression of *groESL* has been found to confer ethanol and butanol tolerance [64], [65].

Given the varied functions encoded by these furfural tolerance genes, and the common reduced lag phase observation, we sought to better understand if these genes were conferring tolerance through previously implicated physiological mechanisms. Specifically, we assessed the effect of overexpression of each of these genes on furfural reduction and DNA mutation rates.

### Increased furfural reduction from lpcA overexpression

**Figure 4:**
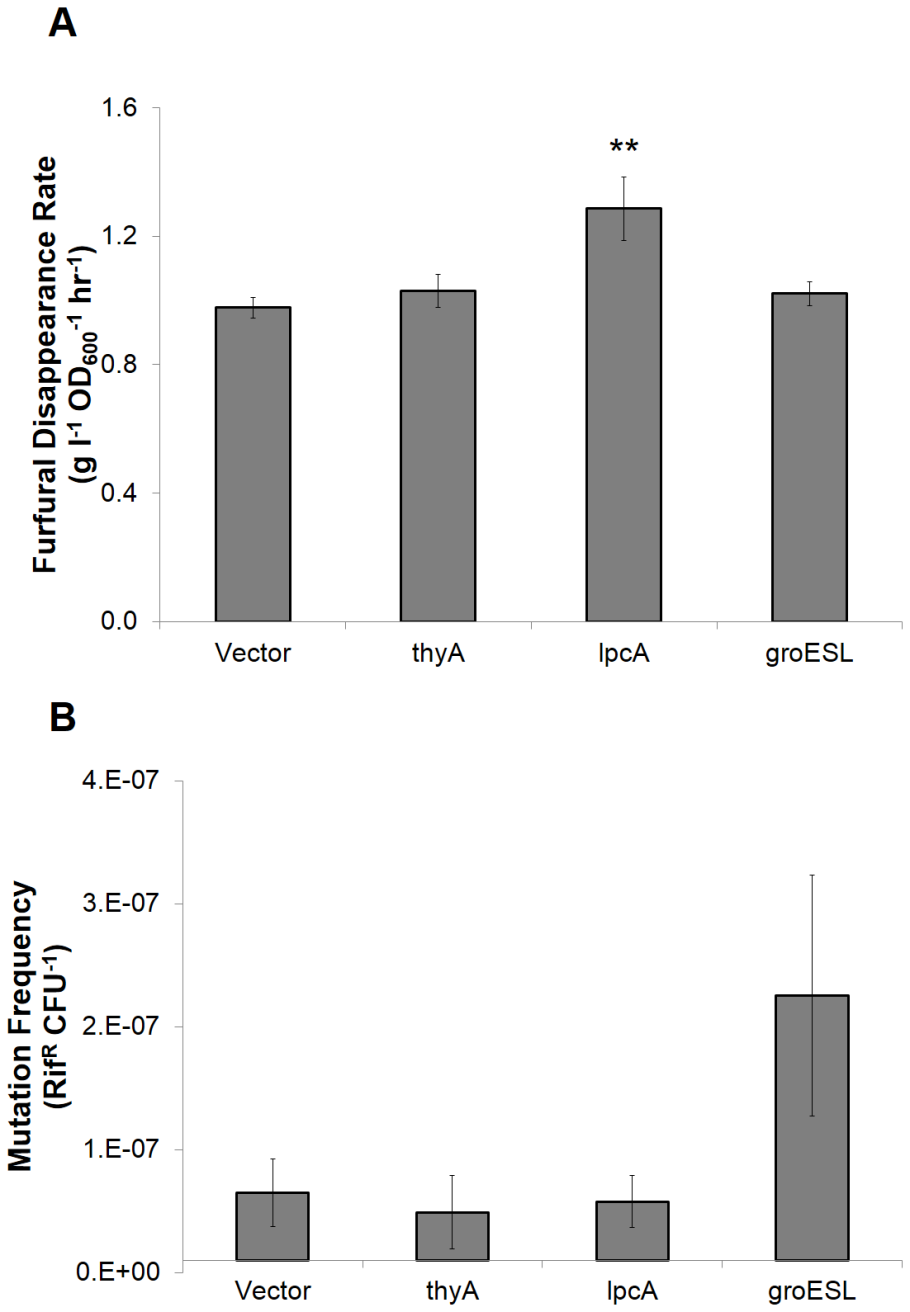
Phenotypic analysis of tolerant clones for furfural reduction and DNA mutation frequency. A) Samples were collected for measuring furfural in growth curve cultures with 0.75 g l^−1^ furfural initial concentration. Furfural concentrations were normalized to cell number (optical density) for each value and disappearance rate was calculated during the transition from lag to exponential phase (n = 3). B) Frequency of rifampin resistance of cells treated with furfural (n = 4). Error bars represent one standard error. Double asterisks denote *p*<0.05.

Furfural is known to be reduced to the less toxic furfuryl alcohol in *E. coli* [29], [66]. This reduction has been primarily linked to the action of a low K_M_ NADPH-dependent oxidoreductase encoded by *yqhD* [32]. It is thought that the increased oxidation of NADPH required for furfural reduction limits the availability of NADPH reducing equivalents that are required for key biosynthetic reactions like sulfur assimilation [30] and nucleotide synthesis [31]. Indeed, for our fastest growing strain in liquid culture, *lpcA*, we measured 32±10% increase in furfural reduction rate compared to control (Fig. 4A). This observation is consistent with our speculation that increased flux through the PPP could lead to elevated NADPH flux and thus increased reduction rates. Neither the *thyA* or *groESL* clones were observed to alter furfural reduction rates.

Furfural was found to induce a significant lag phase longer than cells grown without furfural treatment, which is traditionally linked to the aforementioned NADPH starvation concomitant with furfural treatment [66]. Despite a substantial lag phase, growth during furfural reduction was observed in our growth curve assessments (Fig. 3). Approximately 60–70% of the furfural still remained after 12 hours, roughly coinciding with the onset of exponential phase. All strains had reduced virtually all of the furfural within 20–24 hours (data not shown).

Assessing the redox state and furfural tolerance of cells overexpressing *lpcA*, and other enzymes related to PPP flux, could be a potential path for future research to complement the transhydrogenase overexpression approach recently used [30], [36]. This approach could serve as an alternative to strategies directed at replacing NADPH-dependent oxidoreductase reduction with NADH-dependent oxidoreductases [33], [34], [36].

### Tolerance genes do not alter DNA mutation frequency

Since furfural is a known DNA mutagen [26]–[28], we hypothesized that our furfural tolerance genes might affect DNA mutation frequencies and thereby lead to tolerance. The mutation frequency was measured by treating cell cultures with furfural and then plating with rifampin to measure the number of spontaneous mutants, compared to total viable cells (Fig. 4B) [54], [55]. Surprisingly, no clones exhibited significantly altered DNA mutation frequency from control (*p*>0.05 for all). Although the *groESL* clone did appear to increase mutation frequency ∼3-fold, statistical analysis indicated that this increase was not significant (*p*>0.08).

We had hypothesized that we would observe altered DNA mutation frequency for the *thyA* clone based on its presumed role of increasing dTMP availability required for DNA repair under furfural treatment [31] and for the *groESL* clone due to the chaperone's role in stress response and its ability to stabilize mutated proteins [67]. It is possible that the level of furfural treatment here did not deplete DNA repair pathways enough in order to elicit an observable difference, although previous studies have also indicated that furfural treatment does not always elevate mutation frequencies beyond what native repair mechanism can handle [68]. It is also worthwhile to note that ThyA is involved in formyl-tetrahydrofolate biosynthesis (converting THF to 5,10-methylene-THF during the dUMP to dTMP reaction), which is a pathway previously associated with tolerance to acetate [7] and 3-hydroxypropionic acid [44], and thus might suggest a more general role for ThyA in chemical tolerance beyond pyrimidine biosynthesis and DNA repair. In the case of the *groESL* clone, our data suggest that any role GroESL has in stabilizing mutations that might arise from furfural treatment is not significant, which suggests that GroESL may rather be acting to stabilize wild-type proteins whose function or formation is altered in the presence of furfural.

### Validation that lpcA and groESL overexpression confer furfural tolerance

Because *lpcA* and *groESL* have not been previously identified to confer furfural tolerance, we aimed to verify that our plasmid constructs resulted in increased transcription for the targeted genes. Transcript levels were observed for *lpcA* to be 98±24 fold-increase over the control strain. The *groESL* construct had increased expression of its *groES* and *groEL* genes of 150±86 and 126±48 fold, respectively (Table S3).

**Figure 5:**
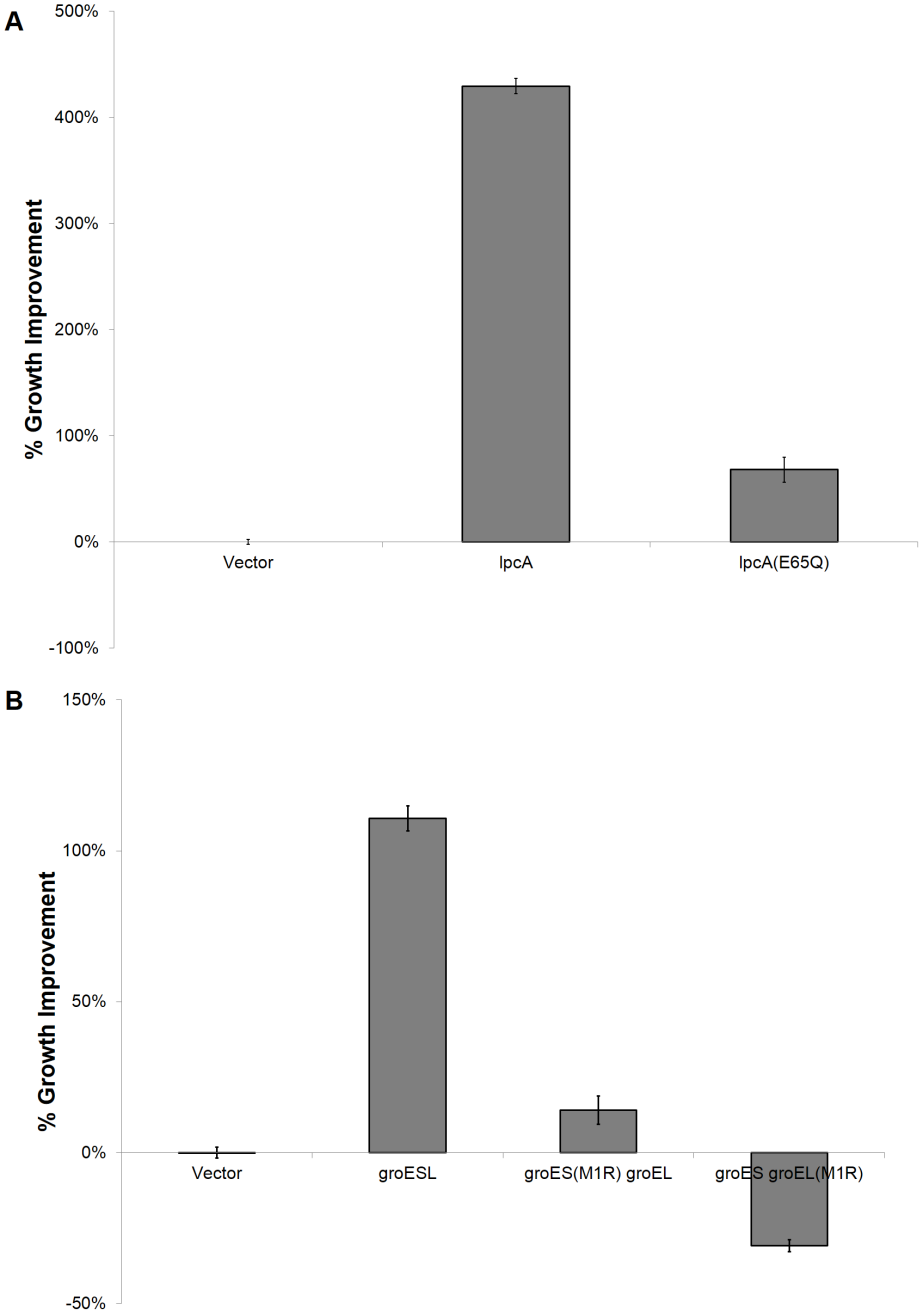
Mutational studies on *lpcA* and *groESL* clones. Mutations were introduced onto the plasmids within the coding sequence targeted for (A) *lpcA* or (B) *groESL*. Cultures were grown in 0.75 g l^−1^ furfural for 20 hr. (n = 3; error bars represent standard error). Percentage improvement was calculated as the difference of the test strain subtracted from the control, divided by the control.

While this data confirmed increased expression from the plasmid based constructs, we further wanted to verify that tolerance was conferred by functional expression of LpcA or GroESL. To do so, we introduced a missense mutation into the coding sequences of ech of these genes. For *lpcA*, we targeted a residue in the active site with the E65Q mutation, which has previously been reported to confer undetectable enzymatic activity [69]. For *groESL*, we replaced the start codon (ATG with CGG for an M1R mutation) of *groES* or *groEL*. When tested for growth in 0.75 g l^−1^ furfural, the *lpcA* plasmid conferred 429±7% improvement in growth over blank vector control, whereas *groESL* conferred 111±4% improvement (Figure 5). The missense mutation clone *lpcA*(E65Q) conferred a slight improvement in tolerance (68±12%; *p*<0.05), which could be a result of low enzymatic activity levels below the threshold of activity of the previous assay [69], but is markedly below the improvement conferred by the wild-type sequence. Additionally, the M1R missense mutation in *groES* conferred no difference in growth compared to blank vector (*p*>0.1), and the M1R missense mutation in *groEL* conferred a decrease in growth (reduction of 30±2%). Taken together, our data suggests that at the expression levels conferred by expression on the pSMART-LCK vector rely on functional expression of the enzyme LpcA enzyme or GroESL complex in order to confer tolerance to furfural.

## Conclusions

Much research has been performed over the past decade to uncover mechanisms of furfural toxicity and to engineer furfural tolerance in *E. coli* [29]–[37], [39], [66]. Here, we used the SCALEs method [13] to not only map fitness effects across the entire *E. coli* genome, but also to identify and confirm both novel (*lpcA* and *groESL*) and previously identified (*thyA* [31]) furfural tolerance genes. We determined that overexpression of *lpcA* increased observed furfural reduction. LPS core formation, for which LpcA plays a part, is vital for tolerance to chemical inhibitors [7], [9], [57]–[59]. To this end, analysis of GO term enrichment from our high-fitness genes suggests that membrane and wall biosynthesis is important for furfural tolerance. Alternatively, *lpcA* overexpression may increase flux through the PPP and thereby increase NADPH availability for furfural reduction. Overexpression of *groESL* also conferred increased growth, but did not alter the rate of furfural reduction or mutation frequency. It is possible that furfural elicits responses similar to those from solvent stress, where *groESL* overexpression has been shown to confer tolerance [64]. ThyA overexpression did not alter DNA mutation frequency even though it has previously been implicated in increasing DNA repair under furfural stress [31].

Robust microbes for lignocellulosic biofuel production must be engineered for multiple functions–production of a desired product, tolerance to feedstock and product, co-utilization of feedstock carbon sources–that all work in concert together. Our study here expands the understanding of furfural tolerance genes and thus provides additional targets for engineering furfural tolerance. Ultimately, finding genetic manipulations that are beneficial to multiple biocatalyst functions will enable rapid, reliable, and improved biofuel production in the future.

## Supporting Information

Figure S1 **Genomic position alignments of library clones for gene fitness assignments.** SCALEs clone fitness scores from the 2, 4, and 8 kb libraries are based on clone frequency with and without selective pressure. The *lpcA* gene is shown in green, with neighboring genes shown in gray.
(PPTX)

Figure S2 **Enriched biological processes GO terms in SCALEs selection.** Yellow boxes represent significantly enriched GO terms and non-significant terms are condensed to nodes. Red arrows connect two significantly enriched GO terms, whereas black arrows connect a non-significantly enriched term (node) to a significantly enriched term (yellow box). Analysis was performed with the Batch Genes GOEAST online tool as described in the text.
(PPTX)

Table S1 Primers used in creating SCALEs target clones.
(DOCX)

Table S2 Enriched genes from the furfural SCALEs selection.
(XLSX)

Table S3 Fold-change expression of targeted genes from Control for pLPCA and pGROESL plasmid constructs.
(DOCX)
