# Supplementary figures and images for: Genome-Wide Mapping of Furfural Tolerance Genes in Escherichia coli
Source: PLoS One. 2014 Jan 28;9(1):e87540. doi: 10.1371/journal.pone.0087540 (PMC3905028; doi:10.1371/journal.pone.0087540)

## Slide 1
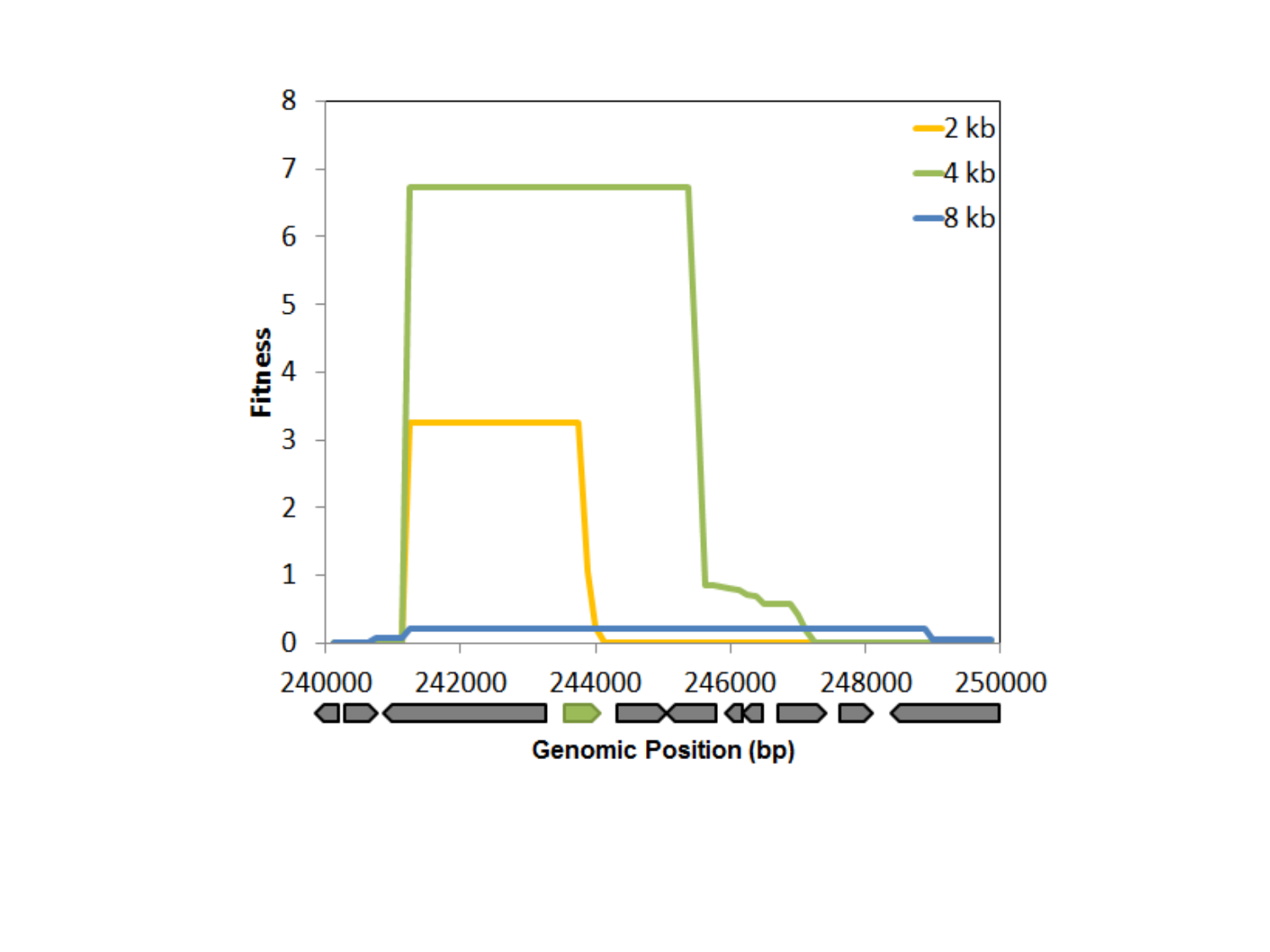

Supplement: Figure S1 — Genomic position alignments of library clones for gene fitness assignments. SCALEs clone fitness scores from the 2, 4, and 8 kb libraries are based on clone frequency with and without selective pressure. The lpcA gene is shown in green, with neighboring genes shown in gray. (PPTX) [file pone.0087540.s001.pptx]

## Slide 1
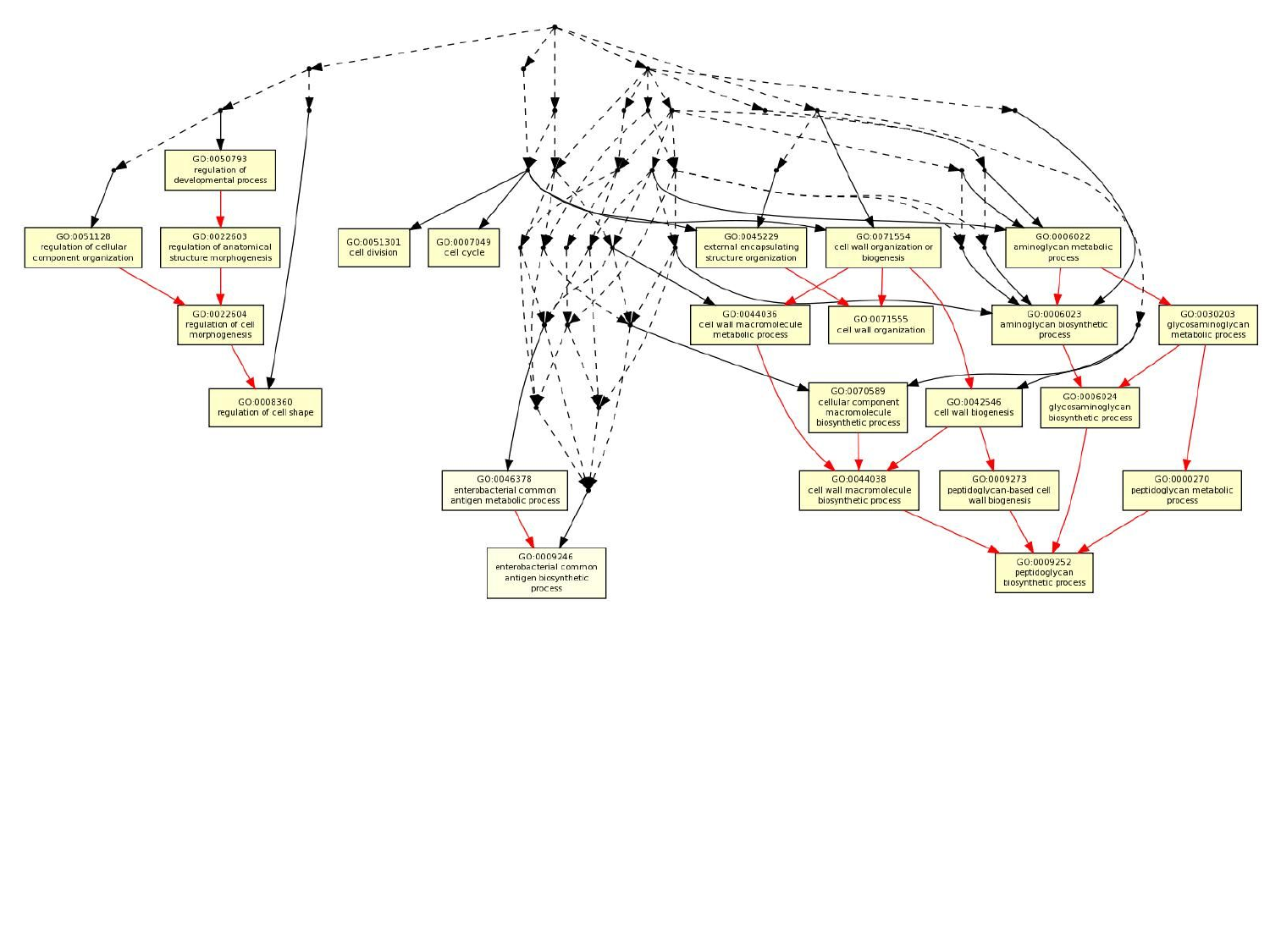

Supplement: Figure S2 — Enriched biological processes GO terms in SCALEs selection. Yellow boxes represent significantly enriched GO terms and non-significant terms are condensed to nodes. Red arrows connect two significantly enriched GO terms, whereas black arrows connect a non-significantly enriched term (node) to a significantly enriched term (yellow box). Analysis was performed with the Batch Genes GOEAST online tool as described in the text. (PPTX) [file pone.0087540.s002.pptx]
