# Supplementary material for: Genome-Wide Mapping of Furfural Tolerance Genes in Escherichia coli
Source: PLoS One. 2014 Jan 28;9(1):e87540. doi: 10.1371/journal.pone.0087540 (PMC3905028; doi:10.1371/journal.pone.0087540)
Supplement: Table S1 — Primers used in creating SCALEs target clones. (DOCX) [file pone.0087540.s003.docx]

**Table S1.** Primers used in creating SCALEs target clones

| **Clone_Direction** | **Sequence (5’-3’)** |
| --- | --- |
| thyA_for | CGTTGCAAAATTTCGGGAAGGCGT |
| thyA_rev | GCTGCTGCTGGAAGGTGTGGT |
| ybiY_rev | ATGCGGTCGCTGAGCGTGTC |
| ybiY_rev | CCTGGGCAAACAGACGCCCC |
| groESL_for*^a^* | GAGACCGGAATTCCGGTGACGGCGATGAAGAAATTGCGA  GAGACCGGAATTCCGGACATTTCTGCCCGGGGGTTTGT |
| groESL_rev*^a^* | GAGACCGGAATTCCGGACATTTCTGCCCGGGGGTTTGT |
| lpcA*^b^*_for | AAGCCCCTTACTTGTAGGAGGTCTGA |
| lpcA*^b^*_rev | TCGCATCAGGCATCAGCGCACAAAT |
| ybaK_for | GCCGCTGGATGTGAGTGTTT |
| ybaK_rev | AAGCGACGGTGTAACTCGAT |

*^a^* Due to the large size of this insert, it was constructed using cohesive-end cloning and contains the EcorI site.

*^b^lpcA* plasmid was constructed by Woodruff et al [[1](#_ENREF_1)].

**Reference**

1. Woodruff LB, Pandhal J, Ow SY, Karimpour-Fard A, Weiss SJ, et al. (2013) Genome-scale identification and characterization of ethanol tolerance genes in Escherichia coli. Metab Eng 15: 124-133.
