# Supplementary material for: Genome-Wide Mapping of Furfural Tolerance Genes in Escherichia coli
Source: PLoS One. 2014 Jan 28;9(1):e87540. doi: 10.1371/journal.pone.0087540 (PMC3905028; doi:10.1371/journal.pone.0087540)
Supplement: Table S3 — Fold-change expression of targeted genes from Control for pLPCA and pGROESL plasmid constructs. (DOCX) [file pone.0087540.s005.docx]

**Table S3.** Fold-change expression of targeted genes from Control for pLPCA and pGROESL plasmid constructs.

|  | ***lpcA*** | ***groES*** | ***groEL*** |
| --- | --- | --- | --- |
| **pLPCA** | **98 ± 24** | **N/A** | **N/A** |
| **pGROESL** | **N/A** | **150 ± 86** | **126 ± 48** |
